# A gene expression profile test to resolve head & neck squamous versus lung squamous cancers

**DOI:** 10.1186/1746-1596-8-44

**Published:** 2013-03-11

**Authors:** Anita Lal, Rebecca Panos, Mira Marjanovic, Michael Walker, Eloisa Fuentes, Gregory J Kubicek, W David Henner, Ljubomir J Buturovic, Meredith Halks-Miller

**Affiliations:** 1 Pathwork Diagnostics, 595 Penobscot Dr, Redwood City, CA 94063, USA; 2 Department of Radiation Oncology, University of Pittsburgh, Pittsburgh, PA 15232, USA

**Keywords:** Diagnostic test, Gene expression, Tissue of origin, Lung cancer, Head & neck cancer

## Abstract

**Background:**

The differential diagnosis between metastatic head & neck squamous cell carcinomas (HNSCC) and lung squamous cell carcinomas (lung SCC) is often unresolved because the histologic appearance of these two tumor types is similar. We have developed and validated a gene expression profile test (GEP-HN-LS) that distinguishes HNSCC and lung SCC in formalin-fixed, paraffin-embedded (FFPE) specimens using a 2160–gene classification model.

**Methods:**

The test was validated in a blinded study using a pre-specified algorithm and microarray data files for 76 metastatic or poorly-differentiated primary tumors with a known HNSCC or lung SCC diagnosis.

**Results:**

The study met the primary Bayesian statistical endpoint for acceptance. Measures of test performance include overall agreement with the known diagnosis of 82.9% (95% CI, 72.5% to 90.6%), an area under the ROC curve (AUC) of 0.91 and a diagnostics odds ratio (DOR) of 23.6. HNSCC (N = 38) gave an agreement with the known diagnosis of 81.6% and lung SCC (N = 38) gave an agreement of 84.2%. Reproducibility in test results between three laboratories had a concordance of 91.7%.

**Conclusion:**

GEP-HN-LS can aid in resolving the important differential diagnosis between HNSCC and lung SCC tumors.

**Virtual Slides:**

The virtual slide(s) for this article can be found here: http://www.diagnosticpathology.diagnomx.eu/vs/1753227817890930

## Background

Metastatic squamous cell carcinomas of the head & neck (HNSCC) and squamous cell carcinomas of the lung (lung SCC) appear similar on microscopic examination and are often indistinguishable using traditional histopathology. While immunohistochemical approaches are very useful in distinguishing squamous cell carcinomas from other carcinomas such as adenocarcinomas, they fail to clearly identify the site of origin of the squamous cell carcinoma [1]. Both HNSCC and lung SCC show positive immunoreactivity with squamous cell carcinoma markers such as p63 and CK5/6 [2].

Further confounding this diagnostic dilemma is the fact that head & neck cancers and lung cancers often occur in the same patient. Both cancers have similar etiologies and risk factors such as tobacco use [3,4]. Patients with a prior laryngeal cancer also have a 4.5-fold increased incidence ratio of lung cancer when followed for >5 years [5]. Thus, in patients with a prior history of head & neck cancers, a new lung lesion might represent a new primary lung cancer or may represent metastasis from the previously treated head & neck cancer [6,7].

The distinction between HNSCC and lung SCC, and in particular the distinction of metastatic versus primary cancers for lung lesions, is important for optimal clinical management of patients. Prognosis and therapeutic options for patients with metastatic head & neck cancer are considerably different from those for patients with a localized secondary lung cancer. While metastatic head & neck cancer patients have an expected median survival of 10 months to a year, patients with solitary lung cancer have a median survival of 48 months [8,9]. Similarly, therapeutic strategies for metastatic head & neck cancer patients are markedly different from therapeutic strategies for patients with solitary lung cancer lesions [3]. While patients with primary lung cancers are more likely to receive lung lobectomies, associated with a 3% mortality rate, adjuvant chemotherapy, and other aggressive forms of therapy, metastatic head & neck cancer patients are more likely to be treated with palliative chemotherapy alone [10,11].

Molecular diagnostic tests that use gene expression profiling with microarrays to classify cancers according to their primary sites are now a feasible tool for cancer diagnosis [12-16]. Advances in gene annotation and array design along with the use of standardized protocols and array platforms across laboratories have made microarray-based gene expression profiling highly reproducible [13,16-19]. These assays have the advantage of measuring the expression of a multitude of biomarkers simultaneously. Additionally, the use of RNA from formalin-fixed paraffin embedded (FFPE) tissue in microarray-based diagnostics has become more common, considerably expanding the utility of these diagnostic tests [13,16,20-22].

Gene expression profiling has previously been used in several studies to distinguish head & neck carcinomas from clinically normal tissues [23-26]. These data have been used to develop predictive models that discriminate oral squamous cell carcinomas from normal specimens or distinguish dysplasia from normal tissue. These models have been validated in independent sets of samples and shown to have high sensitivity and specificity. In contrast, studies that use gene expression profiling to distinguish between head & neck cancers and lung cancers have been rare [27]. One prior study showed that histologically similar squamous cell carcinomas have distinct gene expression profiles based on their anatomic sites of origin [27]. While this profile was not validated using an independent set of samples, it does appear that it is feasible to classify squamous cell carcinomas based on their primary sites using gene expression.

In order to better clarify HNSCC versus lung SCC, we have developed a gene expression based diagnostic test, GEP-HN-LS (Pathwork® Tissue of Origin Head & Neck Test, Pathwork Diagnostics, Inc., Redwood City, CA, USA), that can be used to aid in the differential diagnosis of squamous carcinomas of the head & neck and lung in FFPE tissue. For patients with HNSCC who develop a lung nodule, a test differentiating second primary versus metastatic disease would have significant clinical utility. Patients with a second primary have a high chance at cure with aggressive therapy while patients with metastatic disease are not curable and are best treated with palliative chemotherapy.

## Methods

### FFPE tumor specimens

FFPE tumor specimens for the validation study were acquired from seven different human tumor tissue banks using Institutional Review Board-approved procedures. All specimens were excisional biopsies and had a known clinical diagnosis of lung SCC or HNSCC cancer based on histopathology and clinical history. All specimens had a known biopsy site and were either metastatic tumors or primary tumors that were poorly differentiated or undifferentiated. The anatomical sites of the primary HNSCC cases were larynx, oral cavity, nasopharynx and oropharynx. Esophageal carcinomas were not an included morphology. Other available clinical information that was recorded for each specimen included age, race, tumor grade, cancer stage, tissue dimensions and date (year) of resection. Further details regarding specific specimen characteristics are included in the results. H&E sections adjacent to the tumor sample were reviewed by a pathologist to determine the percentage of tumor tissue (including stroma), normal tissue and necrosis [28]. The pathologist assessed the tumor content by microscopic examination of cases where the percent tumor was estimated to be ≥80%. If less than 80% by visual inspection, digital photomicrographs were made using a Zeiss Axiocam with AxioVision software mounted on a Zeiss AxioScope A1 microscope. The tumor and non-tumor areas were digitally outlined and the software calculated the areas of each. Then, percent tumor was calculated. The GEP-HN-LS test has a specimen entry requirement of at least 60% tumor tissue including intra-tumoral stroma [28]. Therefore, only specimens that met this minimal tumor content quality criterion were included in this study. The set of specimens used for algorithm training was completely independent from the set of specimens used for clinical validation. Microarray data for clinical validation specimens can be found at Gene Expression Omnibus (GSE44177).

### RNA extraction, target preparation, and microarray processing procedures

FFPE tumor specimens were processed as described in a previous study [13,16]. Briefly, total RNA was isolated from 10 μm thick FFPE sections using the Agencourt FormaPure system (Beckman Coulter Genomics, Beverly, MA) and the Ambion DNase I RNA-free kit (Life Technologies, Austin, TX). Total RNA concentration was assessed by spectrophotometry (OD 260 nm, NanoDrop, Wilmington, DE), and the purity was judged by the ratio of absorbance at 260 nm to 280 nm (A_260_/A_280_). Thirty ng of total RNA was amplified using the RampUp kit (Genisphere, Hatfield, PA) to generate biotin-labeled cDNA. Labeled cDNA was hybridized to a Pathchip® microarray (manufactured by Affymetrix, Santa Clara, CA), and washed and stained using commercially available reagent kits and protocols (Affymetrix, Inc., Santa Clara, CA). The arrays were scanned using either the Affymetrix GCS3000Dx system or the Affymetrix 7G Scanner controlled by the Affymetrix AGCC software. The resulting raw intensity microarray data files were analyzed for data quality.

GEP-HN-LS includes several processing quality metrics for sample processing to continue and for a test result to be reported out. Firstly, a minimum total RNA yield of 30 ng at a concentration of ≥9.5 ng/μl and with an A_260_/A_280_ ratio of ≥1.0 was required to proceed to target preparation. A minimum yield of 2.5 μg of labeled cDNA was required to proceed to microarray analysis. Finally, microarray data quality is verified to meet prespecified quality control metrics of Overall Signal ≥ 10, Percent Present ≥ 5, and Regional Discontinuity ≤ 0.84, calculated as described previously [13,16].

### Specimen processing sites

All specimen preparation and processing was performed at one of three independent processing laboratories: Pathwork Diagnostics Laboratory (Redwood City, CA), Expression Analysis (Durham, NC) and GeneLogic, Inc (Gaithersburg, MA). Pathwork Diagnostics Laboratory and GeneLogic, Inc processed specimens for the clinical validation study; inter-site reproducibility was assessed at Pathwork Diagnostics Laboratory (Site #1), Expression Analysis (Site #2) and GeneLogic, Inc (Site #3); intra-site repeatability (precision) was assessed at Pathwork Diagnostics Laboratory. Laboratories performing the test were blinded to the known clinical diagnosis. All microarray data files were transferred to Pathwork Diagnostics for data quality assessment and analysis through GEP-HN-LS.

### Algorithm development

The GEP-HN-LS test standardization algorithm was developed by evaluating >5000 tissue specimens from a range of tissue types that were processed at 11 laboratories. The classification algorithm was developed using a database of 249 lung SCC and 242 HNSCC specimens that had a known lung SCC or HNSCC cancer diagnosis based on clinical history. Similar to the validation specimens, the training set specimens were a mixture of primary and metastatic cancers with the majority of the cases being primary cancers. In contrast to the validation specimens, where the primary cancers were restricted to poorly differentiated and undifferentiated cases, the training set included a large number of well and moderately differentiated cancers. Among the metastatic cases, lymph node was the primary biopsy site. Other biopsy sites included brain, abdominal wall and dermal tissues. A machine learning approach was used to select the optimal model for classifying lung SCC and HNSCC cancers. To minimize overfitting in the presence of a high number of predictors and a relatively small training set, we applied nested cross-validation approach, which is to the best of our knowledge state-of-the-art method in the context of small sample size/high dimensional problems common in genomics [29]. Validation results on the independent clinical set of previously unseen specimens confirmed that the method adequately prevented excessive overfitting. The optimal model consisted of a list of 2600 probe-sets (2160 independent genes) and a set of coefficients that are combined to produce 2 Similarity Scores. The 2 Similarity Scores correspond to the probability that the gene expression profile of the input specimen matches the expression profile of lung SCC and HNSCC cancers. The standardization and classification algorithms were locked prior to initiation of clinical validation studies.

### Test report

**Figure 1:**
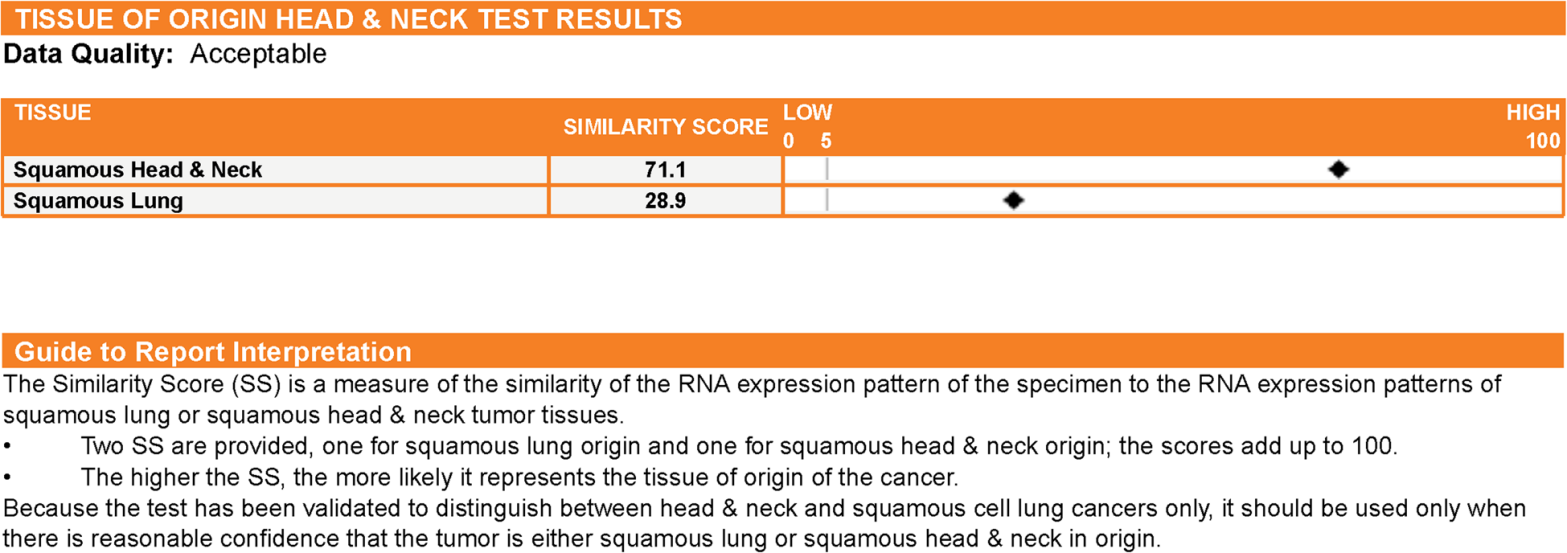
**A sample GEP-HN-LS Test report.** The report presents 2 Similarity Scores, one for head & neck squamous cell cancer (HNSCC) and one for lung squamous cancer (lung SCC) cancers. The two Similarity Scores add up to 100. The tissue type with the higher Similarity Score is the more likely tissue of origin. In the sample shown, HNSCC cancer with a Similarity Score of 71.1 is the more likely tissue of origin.

The Test report (Figure 1) is interpreted using the following guide to report interpretation: The Similarity Score (SS) is a measure of the similarity of the RNA expression pattern of the specimen to the RNA expression patterns of lung SCC and HNSCC tumor tissues. Two SS are generated, one for lung SCC origin and one for HNSCC origin. The scores add up to 100. The higher the SS, the more likely it represents the tissue of origin of the cancer.

### Clinical validation study design

GEP-HN-LS clinical validation study used Bayesian adaptive analysis to determine the design plan and sample sizes [30,31]. Details regarding the clinical validation study design and sample size estimates are provided in Additional file 1: Methods and Table S1. Briefly, the study was designed to have no greater than 5% Type I error and at least 98% power to meet prespecified acceptance criteria. The criteria are based on the mean positive percent agreement (mean PPA) between the Test prediction and the available clinical diagnosis. The acceptance criteria were: mean PPA must be at least 80% and the lower bound of the 95% credible interval must be at least 65%. These requirements were derived from analyses of the training data, requirements for clinical utility of the test, and practicality of obtaining sufficient number of specimens. The study design used three phases, commonly referred to as Looks. Look 1 used one half of the maximum sample size; Look 2 used three-quarters of the maximum sample size; and Look 3 used the maximum sample size. The study design allowed for early termination based on intermediate analyses after each look. Study termination at each look could occur if the acceptance criteria were met or if the predicted probability of success at the maximum sample size, based on the data accumulated up to that point, was less than 5%.

### Reproducibility study design

The intra-site (precision) and inter-site reproducibility of GEP-HN-LS was assessed as follows: For intra-site reproducibility (precision), three adjacent 10 μm thick sections from FFPE tissue blocks of 16 specimens (lung SCC = 8; HNSCC = 8) that gave an agreement with the known clinical diagnosis were processed simultaneously within the same run. For inter-site reproducibility, three adjacent 10 μm thick sections from FFPE tissue blocks of 30 specimens (lung SCC = 15; HNSCC = 15) that gave an agreement with the known clinical diagnosis were processed at each of the three laboratories in this study. Concordance in GEP-HN-LS results obtained from pairwise comparisons of the three sections was used as a measure of test reproducibility for both the intra-site and inter-site analysis. Details regarding sample size estimates for the reproducibility study are provided in Additional file 1: Methods.

### Data analysis

Mean PPA and credible intervals are Bayesian parameters that were used as the primary endpoint for the GEP-HN-LS clinical validation study (see Additional file 1: Methods for details). Performance of the Test was measured as the positive percent agreement (PPA) which is defined as the percent agreement between the GEP-HN-LS test result and the known clinical diagnosis. A receiver operator curve was plotted and AUC was calculated using SigmaPlot 12 [32]. The diagnostic odds ratio was calculated to provide a single indicator of test performance as described earlier [33]. For intra-site and inter-site reproducibility, results were considered concordant if the GEP-HN-LS test results from one section or site matched the result from another section or site. In both cases, an overall pairwise percent concordance in GEP-HN-LS test results is reported. Kappa statistics for agreement were calculated for pairwise comparisons using R Version 2.11.1 (2010-05-31) [34].

## Results

### The GEP-HN-LS test

The GEP-HN-LS test indicates whether HNSCC or lung SCC is the more likely tissue of origin. The test relies on two distinct algorithms, one for standardization and one for classification. The standardization algorithm is used to normalize the raw probe-level intensity values of the gene expression profiles under analysis and reduce technical variation incurred by different processing conditions. The standardized expression values for each probe-set generated by the standardization algorithm are used by the GEP-HN-LS classification algorithm. The classification model measures the expression of 2600 probesets (2160 independent genes) that serve as markers during classification of lung SCC and HNSCC cancers; and produce 2 Similarity Scores that correspond to the probability that the gene expression profile of the input specimen matches the expression profile of lung SCC and HNSCC cancers.

The classification markers are empirically selected by machine learning. Nonetheless, they included several genes that have a known function in lung biology (Table 1). The top five probesets were from surfactant proteins that have a biophysical function in the lung and have a role in pulmonary host defense and regulation of inflammation [35,36]. Expression of specific surfactant proteins in squamous cell carcinomas is controlled by DNA methylation and may be involved in lung cancer pathogenesis [37,38].

**Table 1:** Top 20 biomarkers in the GEP-HN-LS test classification algorithm

**Marker rank**	**Probeset ID**	**Gene symbol**	**Gene name**	**Tissue type with higher expression levels**
1	218835_at*	SFTPA2	surfactant protein A2	Lung
2	209810_at*	SFTPB	surfactant protein B	Lung
3	37004_at*	SFTPB	surfactant protein B	Lung
4	214387_x_at*	SFTPC	surfactant protein C	Lung
5	211735_x_at*	SFTPC	surfactant protein C	Lung
6	211024_s_at*	NKX2-1	NK2 homeobox 1 (TTF-1)	Lung
7	205927_s_at	CTSE	cathepsin E	Lung
8	205654_at	C4BPA	complement component 4 binding protein, alpha	Lung
9	209351_at	KRT14	keratin 14	Head & Neck
10	205916_at	S100A7	S100 calcium binding protein A7	Head & Neck
11	205778_at	KLK7	kallikrein-related peptidase 7	Head & Neck
12	211538_s_at	HSPA2	heat shock 70 kDa protein 2	Head & Neck
13	206697_s_at	HP	haptoglobin	Lung
14	209616_s_at	CES1	carboxylesterase 1	Lung
15	211429_s_at	SERPINA1	serpin peptidase inhibitor, clade A (alpha-1 antiproteinase, antitrypsin), member 1	Lung
16	213553_x_at	APOC1	apolipoprotein C-I	Lung
17	204424_s_at	LMO3	LIM domain only 3 (rhombotin-like 2)	Lung
18	206008_at	TGM1	transglutaminase 1 (K polypeptide epidermal type I, protein-glutamine-gamma-glutamyltransferase)	Head & Neck
19	218644_at	PLEK2	pleckstrin 2	Head & Neck
20	202017_at	EPHX1	epoxide hydrolase 1, microsomal (xenobiotic)	Lung

*Known to have a role in lung biology.

### Processing of clinical validation specimens

Tissue sections from 80 specimens were processed for total RNA. Thirty nanograms of total RNA is required to perform the GEP-HN-LS Test. All 80 specimens yielded at least 30 ng of total RNA at a concentration of ≥9.5 ng/μl and had a median A_260_/A_280_ ratio of 1.94 (range 1.56-2.60) and were processed further. All 80 specimens yielded at least 2.5 μg of biotinylated cDNA and were hybridized to Pathchip microarrays. Of the 80 specimens, 76 specimens passed pre-specified microarray data quality criteria as described in the Methods Section. In all, 95.0% (76/80) of FFPE specimens processed passed all quality criteria for the GEP-HN-LS Test. Data from these 76 specimens were used in data analysis.

### Performance of GEP-HN-LS

The clinical validation study followed a Bayesian adaptive design. Testing was terminated at Look2 after processing of 76 specimens because the acceptance criteria of mean PPA and lower bound of the 95% credible interval for both lung SCC and HNSCC cancers exceeded pre-specified threshold values at this interim analysis (Additional file 1: Table S2). The positive percent agreement (PPA) and percent non-agreement with 95% confidence intervals for GEP-HN-LS are summarized in Table 2. The overall agreement of the GEP-HN-LS test results with the known clinical diagnosis was 82.9% (95% CI, 72.5% to 90.6%). Lung SCC cancers had a higher PPA of 84.2% compared to HNSCC cancers that had a PPA of 81.6% (Fisher’s exact test, p = 1.000; Table 2).

**Table 2:** Accuracy of GEP-HN-LS

**Known clinical diagnosis**	**Positive percent agreement**	**Percent non-agreement**
	**Percent (ratio)**	**Percent (ratio)**
	**[95% Confidence Interval]**	**[95% Confidence Interval]**
Head & Neck Squamous	81.6 (31/38)	18.4 (7/38)
	[65.7-92.3]	[7.7-34.3]
Lung Squamous	84.2 (32/38)	15.8 (6/38)
	[68.7-94.0]	[6.0-31.3]
Overall	82.9 (63/76)	17.1 (13/76)
	[72.5-90.6]	[9.4-27.5]

A discrepancy analysis was performed to assess the cause of the disagreements of the 13 cases with the reference diagnosis. All available clinical and pathology information for the 13 disagreements and a representative set of cases that gave agreements with the reference diagnosis were reviewed by an independent pathologist who was blinded to the test results. None of the cases could be categorized as ‘definitely not the reference diagnosis’. Thus, the reference diagnosis was as accurate as possible with available clinical data and current diagnostic methods.

**Figure 2:**
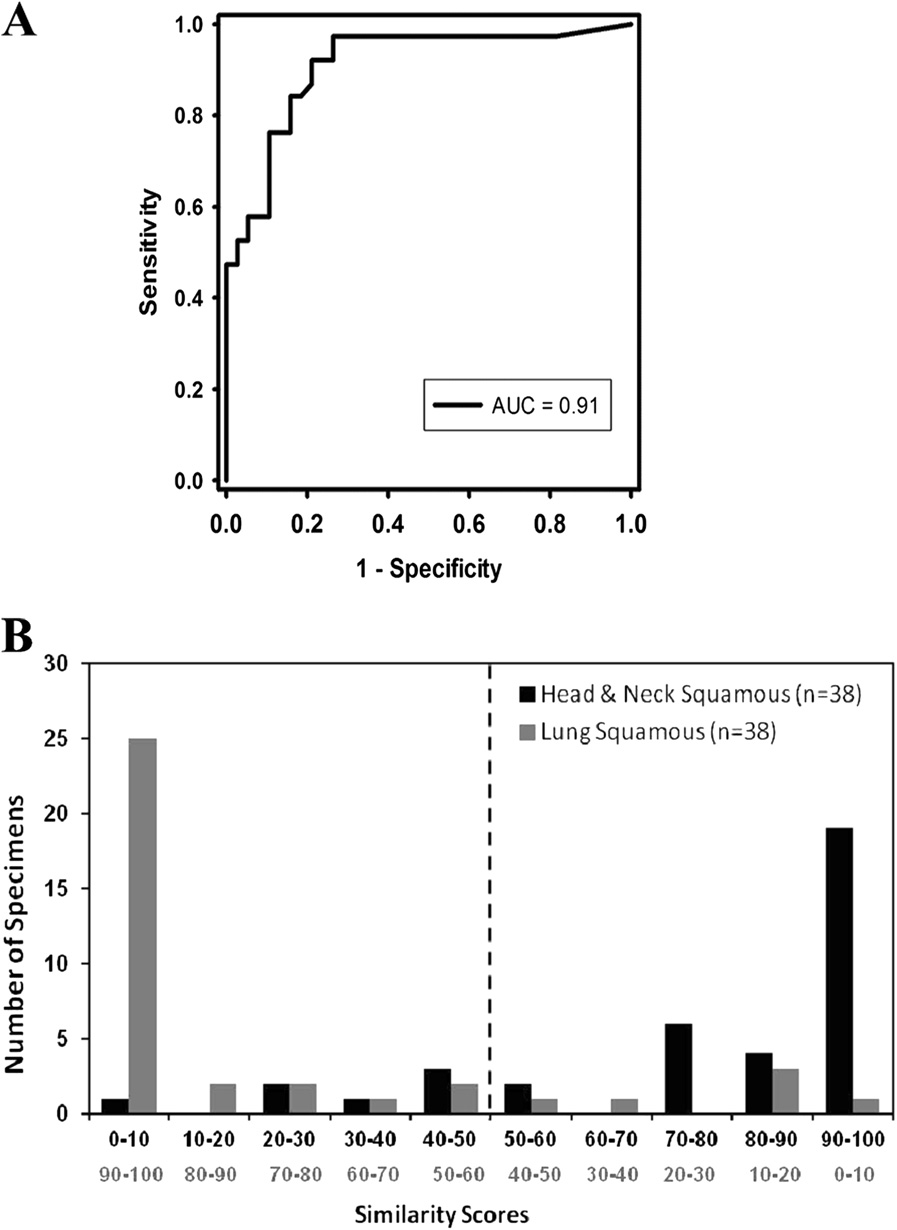
**Performance of GEP-HN-LS.** A receiver operator curve (ROC) curve (**A**) for the GEP-HN-LS Test using results from the independent validation specimens gives an area under the curve (AUC) value of 0.91. Distribution of Similarity Scores (**B**) obtained for the validation specimens show separation of the head & neck squamous cell (HNSCC) cancers and lung squamous cancer (lung SCC) predictions.

An ROC for GEP-HN-LS was plotted and an AUC of 0.91 was obtained indicating high discriminatory performance between lung SCC and HNSCC cancers (Figure 2A). The diagnostic odds ratio of 23.6 (95%CI, 7.1 to 78.2) also indicated that GEP-HN-LS had good discrimination between lung SCC and HNSCC cancers.

A distribution plot of the HNSCC similarity scores in bins of 10 for the 76 clinical validation specimens is plotted in Figure 2B. Since the sum of the similarity scores for HNSCC and lung SCC tissue types is 100, the higher HNSCC similarity scores correspond to lower lung SCC similarity scores and vice versa. The median higher similarity score for cases that gave agreements with the known clinical diagnosis (n = 63) was 96.8 (range, 51.5, 100) for the matched origin and 3.2 (range, 0, 48.5) for the excluded site of origin. In contrast, cases that gave a non-agreement with the known clinical diagnosis (n = 13) had a median higher similarity score for the mismatched origin that was lower at 75.9 (range, 51.1, 100). Sixty one percent (46 of 76) of the specimens assessed gave a similarity score of ≥ 90. For these specimens, the test result indicated the correct HNSCC or lung SCC tissue 95.7% (95% CI: 85.2-99.5) of the time. This also indicates that a similarity score of ≤ 10 rules out that tissue type with a >95% probability (95% CI: 85.2-99.5). Among the HNSCC predictions, 20/37 specimens had a similarity score of ≥ 90 with 19/20 agreements while 26/29 specimens that gave lung SCC predictions had a similarity score of ≥ 90 with 25/26 agreements.

### Performance of GEP-HN-LS according to specimen attributes

The specimens used for clinical validation were acquired in the prior 5 years (Table 3). The age of the specimen did not affect Test performance. For entry into the GEP-HN-LS test, specimens are required to have at least 60% tumor content. The remaining 40% of the specimen could be normal or necrotic tissue. Test performance was consistent at all levels of viable percent tumor above the minimal threshold of ≥ 60% tumor content (Table 3). Specimens in the 60% to 70% tumor content bin had a PPA of 81.8%. The presence of up to 40% necrosis in the specimen under analysis did not diminish performance of GEP-HN-LS (Table 3). In fact, the three specimens in the validation set that had 30 to 40% necrotic content gave agreements with the available clinical diagnosis.

**Table 3:** GEP-HN-LS performance according to specimen attributes

**Specimen attributes**	**No. of specimens**	**PPA**
		**Percent (ratio)**
		**[95% Confidence Interval]**
Age of Specimen		
<1 year	37	78.4 (29/37)
		[61.8-90.2]
1-2 year	8	87.5 (7/8)
		[47.3-99.7]
2-3 year	8	87.5 (7/8)
		[47.3-99.7]
3-4 year	12	83.3 (10/12)
		[51.6-97.9]
4-5 year	11	90.9 (10/11)
		[58.7-99.8]
Percent Tumor Content		
90% < x ≤ 100%	7	85.7 (6/7)
		[42.1-99.6]
80% < x ≤ 90%	19	94.7 (18/19)
		[74.0-99.9]
70% < x ≤ 80%	28	75.0 (21/28)
		[55.1-89.3]
60% ≤ x ≤ 70%	22	81.8 (18/22)
		[59.7-94.8]
Percent Necrosis		
0% ≤ x < 10%	39	82.1 (32/39)
		[66.5-92.5]
10% ≤ x < 20%	19	84.2 (16/19)
		[60.4-96.6]
20% ≤ x < 30%	15	80.0 (12/15)
		[51.9-95.7]
30% ≤ x ≤ 40%	3	100 (3/3)
		[29.2-100]

Abbreviations: PPA, Positive percent agreement; No. of Specimens, Number of specimens in each category.

### Performance of GEP-HN-LS according to patient and cancer attributes

**Figure 3:**
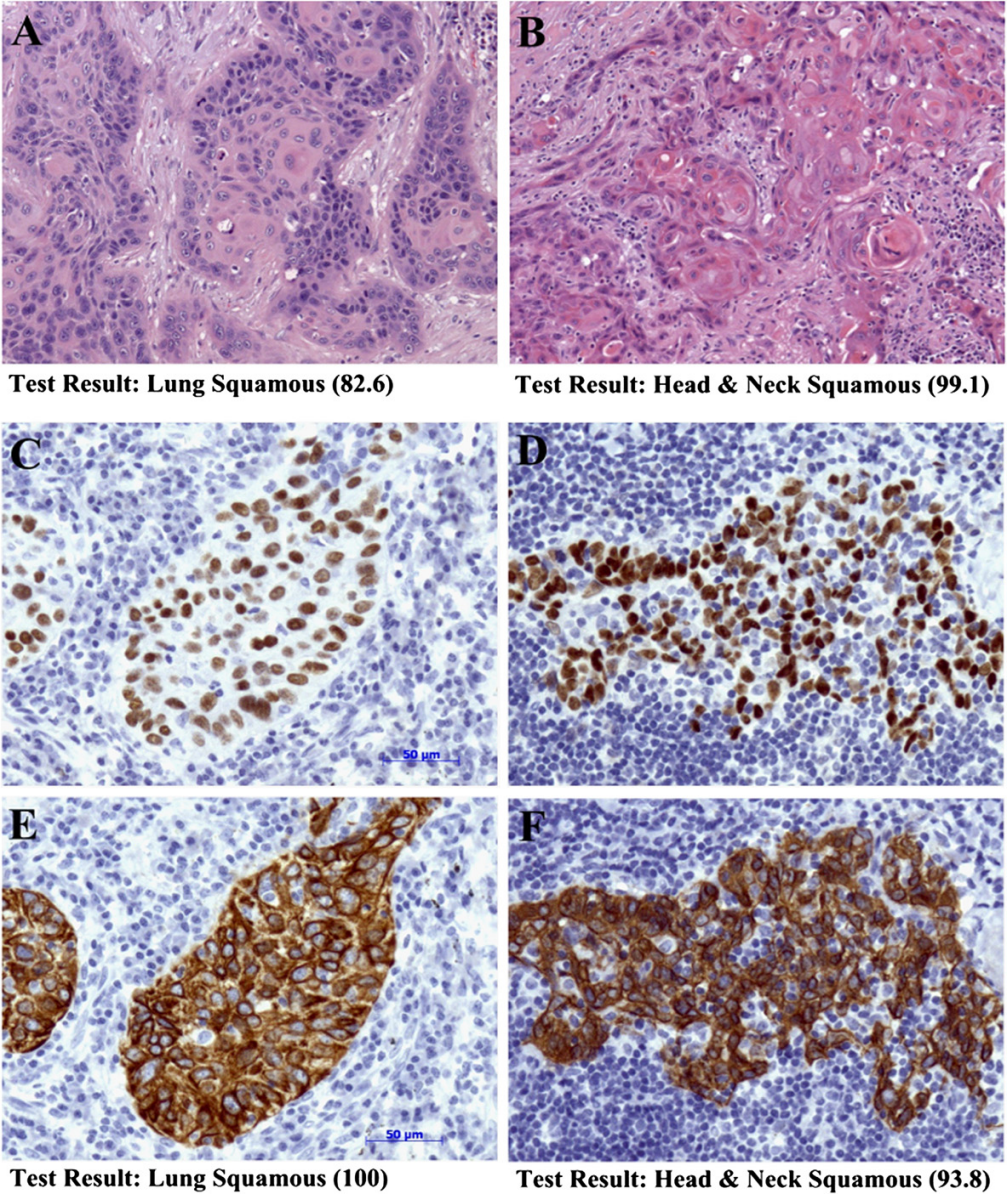
**Distinction of head & neck squamous cell carcinoma (HNSCC) and lung squamous carcinoma (lung SCC) cancers by GEP-HN-LS.** H&E stained sections (**A** and **B**) of a metastatic lung SCC carcinoma (**A**) and a metastatic HNSCC carcinoma (**B**) from the validation specimens show well-differentiated squamous cell morphologic appearance. GEP-HN-LS clearly distinguished between these metastatic tumors. GEP-HN-LS test results were lung SCC with a Similarity Score of 82.6 for the metastatic lung SCC cancer (**A**) and HNSCC with a Similarity Score of 99.1 for the metastatic HNSCC cancer (Photographs **A** and **B** taken at 10X objective magnification). Both poorly differentiated lung SCC (**C, E**) and HNSCC (**D, F**) cancers from the validation specimens show positive immunohistochemical staining for p63 (**C, D**) and CK5/6 (**E, F**), common biomarkers for squamous cell carcinomas. GEP-HN-LS test results for these cancers were lung SCC with a Similarity Score of 100 for the poorly differentiated lung SCC cancer (**C, E**) and HNSCC with a Similarity Score of 93.8 for the poorly differentiated HNSCC cancer (**D, F**) (Photographs **C**, **D**, **E** and **F** taken at 20× objective magnification; Bars equal 50 μm).

The age range of patients from whom validation specimens were obtained was wide. GEP-HN-LS had good performance for all patient age groups (Table 4). The grade of the cancer was known for 70/76 cases included in the validation study. Since primary tumors in the validation set were solely composed of poorly differentiated to undifferentiated cancers, the vast majority of cases were Grade 3 cancers. GEP-HN-LS had an accuracy of 82.1% for these poorly differentiated Grade 3 specimens (Table 4). Representative poorly differentiated HNSCC and lung SCC cases that were indistinguishable by histology and immunohistochemistry but were accurately classified by GEP-HN-LS are shown in Figure 3. The TNM Stage was known for 33/76 cases included in the validation study. GEP-HN-LS performance was equivalent and good for Stage I – II and Stage III – IV cancers. GEP-HN-LS performance was good with both metastatic specimens (80.8%; n = 26) and with poorly differentiated primary tumors (84.0%; n = 50) (Table 4). For metastatic samples, lymph node was the most common biopsy site constituting 80.7% (21/26) of all metastatic cases. Eighty one percent (17/21) of the specimens that metastasized to lymph nodes gave an agreement with the available diagnosis (Table 4). These lymph node metastases included both HNSCC and lung SCC specimens suggesting that GEP-HN-LS could distinguish HNSCC and lung SCC in lymph nodes. The remaining metastatic specimens were biopsied from brain, abdominal wall and dermal tissues. Representative metastatic HNSCC and lung SCC cases harboring well-differentiated squamous cell morphologic appearances are shown in Figure 3. GEP-HN-LS could clearly distinguish between these morphologically similar cancers.

**Table 4:** GEP-HN-LS test performance according to patient and cancer attributes

**Patient/cancer attribute**	**No. of specimens**	**Overall PPA**
		**Percent (ratio)**
		**[95% Confidence Interval]**
Age of Patient (years)		
40-50	16	87.5 (14/16)
		[61.7-98.4]
50-60	25	80.0 (20/25)
		[59.3-93.2]
60-70	25	80.0 (20/25)
		[59.3-93.2]
>70	10	90.0 (9/10)
		[55.5-99.7]
Cancer Grade		
Grade 1	4	100.0 (4/4)
		[39.8-100.0]
Grade 2	10	80.0 (8/10)
		[44.4-97.5]
Grade 3	56	82.1 (46/56)
		[69.6-91.1]
Cancer Stage		
I-II	16	75.0 (12/16)
		[47.6-92.7]
III-IV	17	82.3 (14/17)
		[56.6-96.2]
Primary Versus Metastatic Cancer		
Metastatic	26	80.8 (21/26)
		[60.6-93.4]
Primary	50	84.0 (42/50)
		[70.9-92.8]
Biopsy Sites for Metastatic Cases		
Lymph Node	21	81.0 (17/21)
		[58.1-94.6]
Other	5	80.0 (4/5)
		[28.4-99.5]

Abbreviations: PPA, positive percent agreement; No. of Specimens, Number of specimens in each category.

### Reproducibility of the GEP-HN-LS test

Intra-site reproducibility (precision) was assessed by performing pairwise comparisons of GEP-HN-LS test results obtained from 3 adjacent sections from the same tissue block processed in the same run. Overall percent concordance in test results was 91.4% (Table 5). The median percent coefficient of variation of the similarity score for the known clinical diagnosis was 3.26% (range 0.0 to 64.3). Discordant specimens were outliers and gave the highest percent coefficient of variation. The value of Kappa (κ) statistics was 0.84, 0.81 and 0.81 for the three pairwise comparisons indicating very good (κ ≥ 0.81) agreement in GEP-HN-LS test results between sections processed in the same run.

**Table 5:** Reproducibility of GEP-HN-LS

**Study**	**Concordance**	**Discordance**
	**Percent (ratio)**	**Percent (ratio)**
	**[95% Confidence Interval]**	**[95% Confidence Interval]**
Intra-Site Reproducibility		
Section # 2 versus Section #3	92.3 (12/13)	7.7 (1/13)
	[64.0-99.8]	[0.2-36.0]
Section # 2 versus Section # 4	90.9 (10/11)	9.1 (1/11)
	[58.7-99.8]	[0.2-41.3]
Section # 3 versus Section # 4	90.9 (10/11)	9.1 (1/11)
	[58.7-99.8]	[0.2-41.3]
Overall	91.4 (32/35)	8.6 (3/35)
	[76.9-98.2]	[1.8-23.1]
Inter-Site Reproducibility		
Site # 1 versus Site # 2	90.0 (27/30)	10.0 (3/30)
	[73.5-97.9]	[2.1-26.5]
Site # 1 versus Site # 3	92.6 (25/27)	7.4 (2/27)
	[75.7-99.1]	[0.9-24.3]
Site # 2 versus Site # 3	92.6 (25/27)	7.4 (2/27)
	[75.7-99.1]	[0.9-24.3]
Overall	91.7 (77/84)	8.3 (7/84)
	[83.6-96.6]	[3.4-16.4]

Pairwise comparisons of GEP-HN-LS test results for adjacent sections from 30 specimens processed at three laboratories (PWDL, EA and GLGC) were performed to assess inter-site reproducibility. The percent concordance in test results for PWDL versus EA was 90.0%, for PWDL versus GLGC was 92.6% and for EA versus GLGC was 92.6% (Table 5). The median percent coefficient of variation of the similarity score for the known clinical diagnosis was 3.12% (range - 0.00 to 66.8%). Once again, discordant samples were outliers and gave the highest percent coefficient of variation. Kappa (κ) statistics for agreement for PWDL versus EA was 0.80 (95% CI, 0.56-1.00), for PWDL versus GLGC was 0.85 (95% CI, 0.62-1.00) and for EA versus GLGC was 0.85 (95% CI, 0.63-1.00), indicating good (κ = 0.61 – 0.80) or very good agreement (κ ≥ 0.81) in GEP-HN-LS test results between the three sites.

## Discussion

Squamous cell carcinomas originating in the lung and those originating in the head & neck region are morphologically similar. Additionally, there are no known immunohistochemical markers that can identify the tissue of origin of squamous cell carcinomas. GEP-HN-LS, a novel gene expression profile diagnostic test, can successfully distinguish between lung SCC and HNSCC cancers. Performance was evaluated in an independent set of specimens that were solely composed of either metastatic cancers or poorly differentiated primary cancers. GEP-HN-LS accurately identified the primary site of lung or head & neck in 82.9% of cases with a known clinical diagnosis of squamous cell carcinoma. The majority of specimens had Similarity Scores of ≥ 90 and for these specimens GEP-HN-LS indicated the correct HNSCC or lung SCC tissue 95.7% of the time. Physicians utilizing the GEP-HN-LS test receive both the tissue type result and the similarity score, and can evaluate the confidence of the tissue call based on the similarity score. The data presented in this study support the superiority of GEP-HN-LS in distinguishing HNSCC from lung SCC when compared to human papillomavirus (HPV) testing, which has low sensitivity as it is present in only a subset of HNSCC [26]. Cytokeratin profiling has not been useful in distinguishing between squamous cell carcinomas from various primary sites as squamous cancers have overlapping cytokeratin positivity profiles.

GEP-HN-LS uses 2600 probesets (2160 independent genes) to classify HNSCC and lung SCC cancers. These classification biomarkers were empirically chosen based upon maximal performance observed with the training data and the 20 markers with the highest predictive value included several genes with specific functions in lung biology and possible roles in lung cancer. Surfactant proteins are components of the lipoprotein surfactant complex that reducing surface tension at the alveolar air-liquid interface and facilitate respiratory mechanics [35,36]. They control pulmonary host defense and inflammation, and might have a role in lung cancer pathogenesis [37,38]. Interestingly, NKX2-1 (more commonly known as Thyroid Transcription Factor-1 or TTF-1), a common immunohistochemical marker of lung adenocarcinomas [39,40], was among the top 20 classification markers and had higher expression levels in lung SCC compared to HNSCC cancers. Squamous cell carcinomas are typically negative for nuclear NKX2-1 protein expression [41]. However, cytoplasmic staining of the NKX2-1 protein and expression of the NKX2-1 transcript has been observed in squamous cell carcinomas [42].

Gene expression profiling has previously been successfully used to distinguish between primary lung cancers and metastatic head and neck cancers [27]. The top classification markers identified in the prior study were different from those used by GEP-HN-LS. This is possibly because the prior study utilized frozen tissues and the GEP-HN-LS test is optimized to work on FFPE specimens. In addition, the prior study utilized a different microarray chip and restricted the head & neck cases to those occurring in the oral tongue which is not the most common head and neck cancer subtype [27]. Another study used gene expression profiling to identify differences between head & neck and cervical cancers, the majority of which are also squamous cell cancers [26]. Kallikrien-related peptidase 7, KLK7, was the only marker in our list of top classification markers that was among their differentially expressed genes. Thus, this gene had higher expression levels in HNSCC compared to both lung SCC and cervical cancers.

Gene expression profiling based predictive models that distinguish oral squamous cell carcinomas from normal tissue and oral dysplasia from normal tissue have also been developed [23-26]. The classification markers used by these prior models are distinct from those used by GEP-HN-LS. This is not surprising since the prior models identify the presence of cancer while GEP-HN-LS differentiates between two different cancers.

GEP-HN-LS utilizes FFPE specimens, the most commonly available clinical specimen and success rates for processing FFPE specimens was 95.0%. The use of FFPE specimens along with a high processing success rate is advantageous in the clinical setting, and allows for wide usage of GEP-HN-LS.

The test has been validated for samples that have as low as 60% tumor content. In cases where the tumor content of specimens is <60%, tissue microdissection can be used to enrich the tumor content and achieve the 60% tumor requirement. FFPE specimens used in this study were excisional biopsies. However, FFPE cell buttons from fine needle aspirates (FNA), including bone marrow aspirates, FFPE cell buttons from malignant effusions and FFPE core needle biopsies can be used with GEP-HN-LS as long as they contain ≥ 60% tumor and yield sufficient (≥ 30 ng) total RNA to allow target preparation. Test performance of GEP-HN-LS was consistent regardless of the biopsy site. Both primary and metastatic cases, which have different biopsy sites, performed well with GEP-HN-LS. While our study was not powered to perform sub-population analysis, GEP-HN-LS could distinguish specific cases of HNSCC versus lung SCC that were both in the lymph node. Additionally, test performance for specimens with a lymph node biopsy site was equivalent to specimens with other biopsy sites such as brain or skin.

Since this test has high accuracy and reliability we feel it can be used in a clinical setting. In patients who are newly diagnosed with a solitary lung nodule at the same time as locally advanced HNSCC this test can be used to determine if the patient has two simultaneous primaries or metastatic HNSCC. For patients with two primaries, aggressive therapy for both areas is recommended since cure rates remain high, while metastatic patients should receive only palliative therapies. Similarly, for patients previously treated for HNSCC who on follow-up scans are noted to have a lung nodule, GEP-HN-LS test can be used to determine if this new nodule is metastatic disease or a second primary, for patients with second primaries aggressive surgical resection is indicated.

In this study, we present data validating the accuracy and reproducibility of GEP-HN-LS. The test can be used as an adjunct to histologic and clinical information for difficult to diagnose patients where the distinction between squamous lung cancer and squamous head & neck cancer would impact surgical or drug management of these patients. We anticipate GEP-HN-LS to have particular clinical value for patients with a lung lesion and a history of prior head & neck cancers and for patients with a new diagnosis of HNSCC diagnosed with a concurrent lung lesion. In these cases, the test would aid in distinguishing between a new lung primary or metastatic disease.

## Conclusion

In summary, in the current study, we have validated performance of the GEP-HN-LS test. This test has potential to be a valuable ancillary tool for physicians as they diagnose and make clinical management decisions for head & neck and lung cancer patients.

## Abbreviations

GEP-HN-LS: Gene expression profile test distinguishing Head & Neck Squamous and Lung Squamous cancers; HNSCC: Head & Neck Squamous cell carcinoma; Lung SCC: Lung squamous cell carcinoma; FFPE: Formalin fixed paraffin embedded; FNA: Fine needle aspirates.

## Competing interests

All authors are employees or consultants of Pathwork Diagnostics.

## Authors’ contributions

AL, RP, MM, and LJB participated in conception, study design, conduct of study, data management and analysis and result interpretation. WDH, MHM and GJK participated in conception, study design and result interpretation. EF participated in study design, data analysis and result interpretation. AL authored the manuscript. All authors have reviewed and approved the final manuscript.

## Supplementary Material

Additional file 1 **Methods.** Clinical Validation Study Design and Sample Size Estimates. **Table S1:** Sample Size Estimates and Bayesian Adaptive Design for Clinical Validation of the GEP-HN-LS Test. **Table S2:** Bayesian Performance Characteristics for the Clinical Validation of the GEP-HN-LS Test. Click here for file
