# Supplementary material for: A gene expression profile test to resolve head & neck squamous versus lung squamous cancers
Source: Diagn Pathol. 2013 Mar 11;8:44. doi: 10.1186/1746-1596-8-44 (PMC3614454; doi:10.1186/1746-1596-8-44)
Supplement: Additional file 1 — Methods. Clinical Validation Study Design and Sample Size Estimates. Table S1: Sample Size Estimates and Bayesian Adaptive Design for Clinical Validation of the GEP-HN-LS Test. Table S2: Bayesian Performance Characteristics for the Clinical Validation of the GEP-HN-LS Test. [file 1746-1596-8-44-S1.doc]

**Supplemental Methods**

**Clinical Validation Study Design and Sample Size Estimates**

The clinical validation study uses a Bayesian adaptive design plan that aimed to produce at least 98% power and no greater than 5% Type I error when testing the mean positive percent agreement (mean PPA) with the available clinical diagnosis. The mean PPA was tested using two criteria. Firstly, the mean PPA must be greater than a pre-specified threshold, A, and secondly, the lower bound of the 95% credible interval must exceed a pre-specified lower bound, L. The dual constraint of mean PPA and lower bound of 95% credible interval reduces the likelihood of an inflated Type I error at the multiple interim analyses. The design was set to have a pre-specified maximum sample number with possible early testing termination based on analyses (Looks) at 1/2 and 3/4 of the maximum sample size. Testing termination at each look could occur for two reasons. Firstly, for early success, if the prespecified mean PPA and lower bound of the 95% credible interval thresholds were exceeded and secondly, for futility, if the predicted probability of success at the maximum sample size based on the data accumulated is less than 5%. Within each look, the minimum number of agreements with available diagnosis needed to meet prespecified mean PPA and lower bound of the 95% credible interval thresholds were derived from the posterior probability beta distribution of the PPA with a uniform prior. The posterior distribution becomes p | S,N ~ Beta(1+S, 1+N-S) where S is the number of agreements at the analysis with N samples.

The sample size estimates are summarized in Supplemental Table 1 and were derived using the following input parameters: pre-specified mean PPA, A, was set at 80%; pre-specified lower bound of the 95% credible interval, L, was set at 65%; study power was set at 98%; and the population PPA estimates for head & neck squamous (HNSCC) cancers and lung squamous (lung SCC) cancers were set at 92% and 94% respectively. The population estimates were based on nested cross-validation analyses of the FFPE subset of the training data. In addition, we mandated that at least 25 samples be run for each tissue type.

The first look is performed after processing N1 = 50 specimens (HNSCC=25; lung SCC=25). If the study is not terminated after the first look, a second look is performed after processing an additional N2 = 26 specimens (HNSCC=13; lung SCC=13) for a total of N1+N2 = 76 specimens. If the study is not terminated after the second look, a third and final look is performed after processing an additional N3 = 24 specimens (HNSCC=12; lung SCC=12) for a total of N1+N2+N3 = 100 specimens and the study is terminated. At each look, the number of agreements is compared with the FutilityBound for that look, and the mean PPA and the corresponding 95% credible intervals are calculated and compared to the pre-specified threshold values. The study proceeds to the next look if at least one of the tissue types did not meet the pre-specified threshold values of mean PPA and lower bound of the 95% credible intervals, and both tissue types exceed the FutilityBound requirement. If the number of agreements for either tissue type is lower than the FutilityBound at any look, the study is referred to a cross-functional project team within Pathwork Diagnostics who evaluates whether the study should continue.

**Reproducibility Study Sample Size Estimates**

For both the intra-site and inter-site reproducibility, the lower limit of the 95% confidence interval for a proportion was calculated using the Clopper-Pearson exact one proportion confidence interval in the PASS software. The intra-site reproducibility assessment used a sample size of 16. For the proportion of cases in agreement (concordance), a sample size of 16 produces a one-sided 95% lower-limit confidence interval with a distance from the sample proportion to the lower limit that is equal to 0.213 when the sample proportion is 0.90. The inter-site reproducibility assessment used a sample size of 30. For the proportion of cases in agreement (concordance), a sample size of 30 produces a one-sided 95% lower-limit confidence interval with a distance from the sample proportion to the lower limit that is equal to 0.146 when the sample proportion is 0.87.

**Data Analysis**

The mean value is calculated as the mean of the beta distribution with parameters α=1+S and β=1+N-S, where S=number of agreements and N=total number of samples; mean PPA = α/(α+β); or (1+S)/(2+N). The 95% credible interval, also known as the Bayesian confidence interval, is the interval encompassing 95% of the posterior probability mass.

| **Supplementary Table 1.** Sample Size Estimates and Bayesian Adaptive Design for Clinical Validation of the GEP-HN-LS Test | | | | | |
| --- | --- | --- | --- | --- | --- |
| **Look** | **Sample Size** | **FutilityBound*** | **Pr (Lose)**** | **Pr (Win)***** | **Pr(Type I Error)^$^** |
| **HEAD & NECK SQUAMOUS CANCER** | | | | | |
| 1 | 25 | 18 | 0.3% | 95.5% | 3.2% |
| 2 | 38 | 29 | <0.4% | 98.2% | 3.4% |
| 3 | 50 | 40 | <0.6% | 99.4% | 3.6% |
| **LUNG SQUAMOUS CANCER** | | | | | |
| 1 | 25 | 18 | <0.1% | 98.5% | 3.2% |
| 2 | 38 | 29 | <0.1% | 99.6% | 3.4% |
| 3 | 50 | 40 | <0.1% | 99.9% | 3.6% |
| * FutilityBound is the minimum number of agreements with the available clinical diagnosis needed to proceed to the next Look. **Pr (Lose) is the cumulative percent probability that the number of agreements will be less than or equal to the FutilityBound. ***Pr (Win) is the cumulative percent probability that the acceptance criteria will be met by sample size N. ^$^Pr (Type I Error) is cumulative percent probability of Type I error for the sample size N. | | | | | |

| **Supplementary Table 2.** Bayesian Performance Characteristics for the Clinical Validation of the Tissue of Origin Head & Neck Test | | |
| --- | --- | --- |
| **Known Clinical Diagnosis** | **Mean PPA**  **Percent (ratio)**  **[95% Credible Interval]** | **Mean Percent Non-Agreement Percent (ratio)**  **[95% Credible Interval]** |
| Head & Neck Squamous | 80.0 (31/38)  [66.5-90.7] | 20 (7/38)  [9.3-33.5] |
| Lung Squamous | 82.5 (32/38)  [69.5-92.5] | 17.5 (6/38)  [7.5-30.5] |
| Overall | 82.1 (63/76)  [72.9-89.7] | 17.9 (13/76)  [10.3-27.1] |
